# Digital Biomarkers of Symptom Burden Self-Reported by Perioperative Patients Undergoing Pancreatic Surgery: Prospective Longitudinal Study

**DOI:** 10.2196/27975

**Published:** 2021-04-27

**Authors:** Carissa A Low, Meng Li, Julio Vega, Krina C Durica, Denzil Ferreira, Vernissia Tam, Melissa Hogg, Herbert Zeh III, Afsaneh Doryab, Anind K Dey

**Affiliations:** 1 Mobile Sensing + Health Institute Center for Behavioral Health, Media, and Technology University of Pittsburgh Pittsburgh, PA United States; 2 Information Technology and Electrical Engineering University of Oulu Oulu Finland; 3 Department of Surgery New York-Presbyterian Hospital & Weill Cornell Medical College New York, NY United States; 4 NorthShore University HealthSystem Evanston, IL United States; 5 Department of Surgery UT Southwestern Medical Center Dallas, TX United States; 6 Systems and Information Engineering University of Virginia Charlottesville, VA United States; 7 Information School University of Washington Seattle, WA United States

**Keywords:** mobile sensing, symptom, cancer, surgery, wearable device, smartphone, mobile phone

## Abstract

**Background:**

Cancer treatments can cause a variety of symptoms that impair quality of life and functioning but are frequently missed by clinicians. Smartphone and wearable sensors may capture behavioral and physiological changes indicative of symptom burden, enabling passive and remote real-time monitoring of fluctuating symptoms

**Objective:**

The aim of this study was to examine whether smartphone and Fitbit data could be used to estimate daily symptom burden before and after pancreatic surgery.

**Methods:**

A total of 44 patients scheduled for pancreatic surgery participated in this prospective longitudinal study and provided sufficient sensor and self-reported symptom data for analyses. Participants collected smartphone sensor and Fitbit data and completed daily symptom ratings starting at least two weeks before surgery, throughout their inpatient recovery, and for up to 60 days after postoperative discharge. Day-level behavioral features reflecting mobility and activity patterns, sleep, screen time, heart rate, and communication were extracted from raw smartphone and Fitbit data and used to classify the next day as high or low symptom burden, adjusted for each individual’s typical level of reported symptoms. In addition to the overall symptom burden, we examined pain, fatigue, and diarrhea specifically.

**Results:**

Models using light gradient boosting machine (LightGBM) were able to correctly predict whether the next day would be a high symptom day with 73.5% accuracy, surpassing baseline models. The most important sensor features for discriminating high symptom days were related to physical activity bouts, sleep, heart rate, and location. LightGBM models predicting next-day diarrhea (79.0% accuracy), fatigue (75.8% accuracy), and pain (79.6% accuracy) performed similarly.

**Conclusions:**

Results suggest that digital biomarkers may be useful in predicting patient-reported symptom burden before and after cancer surgery. Although model performance in this small sample may not be adequate for clinical implementation, findings support the feasibility of collecting mobile sensor data from older patients who are acutely ill as well as the potential clinical value of mobile sensing for passive monitoring of patients with cancer and suggest that data from devices that many patients already own and use may be useful in detecting worsening perioperative symptoms and triggering just-in-time symptom management interventions.

## Introduction

Cancer treatments such as chemotherapy and surgery cause a variety of symptoms and side effects that can impair subjective quality of life and functioning. Across a variety of cancer types, fatigue, pain, nausea, and other physical symptoms are highly prevalent and often severe [1,2], and many patients experience multiple symptoms simultaneously [3]. Patients who report more significant symptoms tend to exhibit worse performance status and functional ability [4,5]. Unfortunately, symptoms remain undetected by clinicians up to half of the time [6,7], limiting opportunities for timely and effective clinical management and resulting in undue patient suffering and functional impairment.

Remotely monitoring symptoms *between* hospital or clinic visits may improve our ability to capture severe or bothersome symptoms when they begin to emerge [8]. Smartphones, now owned by 81% of adults and increasing proportions of older adults, those living in rural areas, and all racial groups, offer new opportunities for remote symptom monitoring [9]. Systems leveraging smartphones for real-time patient-reported outcome (PRO) assessment during outpatient chemotherapy have been demonstrated to be feasible [10,11] and to reduce chemotherapy-related morbidity [12]. Although daily PRO symptom data are valuable, long-term assessment of PROs (eg, over months or years of chemotherapy) is burdensome. Indeed, previous work suggests that patients become significantly less compliant at recording symptoms over time [13], with patient compliance dropping to below 50% after 1 month in one longitudinal study [14]. Developing a remote symptom monitoring system that is less reliant on patient compliance may enable longitudinal symptom tracking and management throughout cancer treatment and even after treatment is completed, when symptoms persist for many survivors.

Smartphones are equipped with a rich array of sensors capable of measuring many behavioral and contextual variables, including mobility, location, ambient light and noise, and social interactions [15]. Most users keep their smartphones within arm’s reach at all times and spend over 4 hours per day interacting with the device [16]. Thus, smartphones can gather digital traces as individuals go about their daily routines. From these raw digital data, meaningful behavioral features such as number of unique locations visited, number of outgoing calls placed, and average level of ambient noise detected during the night can be calculated to provide information about behavior patterns in real-world contexts [17].

Smartwatches and other wearable commercial activity monitors are also becoming more widely used, with about 1 in 5 adults using a wearable device [9]. Wearable devices contain sensors such as accelerometers and photoplethysmography which can provide continuous information about activity, sleep, and physiology (eg, heart rate). Together, these mobile sensing technologies enable objective assessment of behavioral patterns that may reflect worsening health status, including severe or increasing symptoms. Moreover, this high-density, multimodal, and objective data collection can be completed with minimal burden to patients; this feature makes this approach highly scalable and appropriate for remotely monitoring patients, even older patients and those who are acutely ill and even over long periods. Given evidence that physical activity and sleep behaviors as well as heart rate have prognostic value in oncology, technology that enables passive quantification of these metrics holds considerable promise for clinical cancer research [18-20].

Applying machine learning classification to smartphone sensor data has been shown to accurately discriminate depressed from nondepressed individuals [21], to recognize depressive and manic episodes in patients with bipolar disorder [22-24], to predict mental health indicators in schizophrenia [25], and to detect binge drinking and other substance use [26]. These methods can also shed light on *which* behavioral features are most useful for detecting or predicting mental health states or risky behaviors. Work applying this approach to passively detect physical health status in patients with cancer is more limited, but results from 14 recent small studies suggest that wearable and smartphone sensor data are related to symptom burden, quality of life, and other clinical oncology outcomes [27].

The perioperative context is an especially critical time for remote patient monitoring, as complications after cancer surgery are common and can escalate into re-admissions that may be preventable if detected and managed earlier. Results from similar studies of patients undergoing surgical oncology procedures found that accelerometer data were useful for quantifying differences in postoperative recovery [28] and for predicting re-admission risk [29]. In this study, we aimed to examine whether smartphone and wearable sensors can be useful in detecting overall patient-reported symptom burden as well as 3 specific physical symptoms (fatigue, pain, and diarrhea) among patients undergoing pancreatic cancer surgery, a complex but potentially curative procedure with postoperative morbidity rates as high as 40% [30].

## Methods

### Participants

Potential study participants were identified for the study by their surgical oncology care team. Men and women aged 18 years or older who were scheduled for pancreatic surgery at a large academic cancer center were eligible and were enrolled at their preoperative clinic visit. Of 72 eligible and approached patients, 60 consented to participate in this study. Surgery was canceled for 4 patients, and 2 withdrew from the study prior to surgery due to poor health or feeling overwhelmed. An additional 10 had insufficient sensor data for analyses based on data cleaning thresholds (described in detail later), leaving 44 participants in our analytic sample (mean age 65.7 years, range 40-82; 41% [18/44] female; 93% [41/44] white). Most patients were undergoing surgery (75% [33/44] robotic, 16% [7/44] open, 9% [4/44] laparoscopic) for pancreatic cancer (36/44, 82%), with the remainder undergoing surgery for benign conditions (eg, pancreatic cysts). Participants were enrolled from January to September 2017.

### Study Procedure

Study assessments began prior to surgery and continued during inpatient recovery after surgery (mean 7-day stay, range 2-22) and for 60 days after postoperative discharge. A total of 13/44 patients (30%) were re-admitted to the hospital at some point during the 60 days. At their preoperative visit, participants were provided with an Android smartphone with the AWARE app installed [31]. AWARE was used to passively collect smartphone sensor data, including movement and approximate location of the phone, device use, metadata about call and SMS events, and ambient light and noise levels. AWARE was also used to collect patient-reported symptom ratings each morning; participants rated the severity of 10 physical and psychological symptoms (pain, fatigue, sleep disturbance, trouble concentrating/remembering things, feeling sad or down, feeling anxious or worried, shortness of breath, numbness or tingling, nausea, diarrhea or constipation) on a scale from 0 (not present) to 10 (as bad as you can imagine). These symptoms were selected because they reflect common core symptoms during oncology treatment [32] and the symptom severity rating format was adapted from the MD Anderson Symptom Inventory [33]. AWARE stored this information on the device and transmitted deidentified data to a secure server over a secure network connection when the device was connected to Wi-Fi. Participants were asked to keep the phone charged and with them at all times and to use the phone for communication as much as possible.

Participants were also given a Fitbit Charge 2 device to wear for the duration of the study, which they were invited to keep after study completion. The Fitbit collected data about activity, sleep, and heart rate. The Fitbit Charge 2 has been shown to measure activity and sleep parameters with acceptable accuracy in older free-living adults [34].

After study completion, participants returned the mobile phones to the study team and received a compensation of US $150. The University of Pittsburgh institutional review board approved all study procedures.

### Data Processing and Analytic Approach

#### Patient-Reported Symptoms

To compute daily symptom burden scores, we summed all 10 symptom ratings to create a composite reflecting total daily symptom burden (mean 15, range 0-97). We then calculated the mean daily symptom burden for each individual patient and then subtracted individual means from each of that patient’s daily symptom burden scores and categorized the resulting residual into average or below average (residual of daily score – individual mean ≤ 0) or high (residual of daily score – individual mean > 0). This approach allowed us to classify each day as a high or low symptom burden day, adjusting for each individual’s typical level of reported symptoms. Approximately 35.99% (487/1353) of all days were classified as high symptom days (proportion of high symptom days for individual patients ranged from ranged from 0% [0/11] to 80% [8/10]). As the data set was imbalanced, we used the support vector machine synthetic minority over-sampling technique (SVM SMOTE) to resample the minority class. We also examined 3 specific physical symptoms (pain, fatigue, and diarrhea because these were the most common in our sample) using a similar approach.

#### Passive Smartphone and Wearable Sensor Data

We computed day-level (24 hours from midnight to midnight) behavioral features from both AWARE and Fitbit data using our Reproducible Analysis Pipeline for Data Streams (RAPIDS) [35]. Accelerometer, activity recognition, application, battery, call, conversation, light, location, SMS text message, and screen features were extracted from AWARE data. Heart rate, step, and sleep features were extracted from Fitbit data. For sleep, features were extracted for any sleep episodes that ended on that day to capture both overnight main sleep and naps. In total, we extracted 213 features from smartphone and Fitbit data; feature descriptions can be found in RAPIDS documentation [35,36]. We also included 3 additional features judged to be important for symptom prediction: (1) days since surgery, because symptoms tended to considerably increase immediately after surgery and then decline over time; (2) most recent symptom burden score, given that high symptom burden scores today tended to predict high symptom burden tomorrow; and (3) participant’s average symptom burden score up to current time point, given the substantial between-participant variability in the range of symptom severities reported. Because symptom ratings were completed each morning, sensor data were used to predict the next day’s symptom burden class.

We dropped sensor and symptom data from the date of surgery (as devices were with caregivers while patients were in the operating room) and from days that the patient was hospitalized (both after surgery and during any subsequent re-admissions, as we anticipated behavioral patterns to differ systematically in the hospital and we are most interested in detecting symptoms when patients are not in a health care setting).

To clean data, we first excluded days with less than 20 hours of sensor data and participants with fewer than 5 days of sensor data. We then dropped features missing more than 30% of values (days) or with 0 variance as well as days missing more than 30% of values (features). We merged sensor data with high/low symptom labels, then again filtered out participants with less than 5 days of valid labeled sensor feature data. After data cleaning, we had 1353 (mean 30.75, range 5-67 per patient) days of sensor data including 142 features from 44 patients.

On average, participants were missing 7.25% of data values (range 0%-19.08%). For each participant, we imputed continuous missing data as follows: (1) missing features in the training set (ie, subset of data used to train the model) were replaced with the average of the 2 closest days; (2) missing features in the test set (ie, subset of data used to evaluate model performance) were replaced with the last valid day’s feature from the training set; and (3) if a participant is missing a specific feature, replace it with the average from the rest of the participants’ data. We imputed categorical missing data as follows: (1) missing features were replaced with the mode of that participant’s training data; (2) if a participant is missing a specific feature, replace it with the mode of the remaining participants’ training data.

Categorical features were converted into integer representation via one-hot encoding. Because the scale of features will not influence the results of tree-based algorithms (eg, light gradient boosting machine [LightGBM]), we normalized numerical features with either min–max, z-score, or scikit-learn package’s robust scaler for the rest of the models. A total of 75 features were selected via mutual information.

We evaluated a number of different binary classifiers, including logistic regression, k-nearest neighbors, support vector machine, random forest, gradient boosting, extreme gradient boosting, and LightGBM. Model performance (ability of the model to generate predicted binary class labels [0 vs 1] that match true class labels) was compared with several baselines: majority class, random weighted classifier, and decision tree using days since surgery, most recent score, and average score (ie, the 3 nonsensor features used in our models). We used nested cross-validation. Three-fold cross-validation was considered for the inner loop to tune hyperparameters and leave-one-day-out cross-validation was considered for the outer loop to evaluate performance and calculate accuracy, precision, recall, F1, and area under the receiver operating characteristic curve (AUC) across all folds. Because our ultimate goal is real-time clinical implementation of these algorithms, we trained models only on past data from that participant as well as data from other participants (ie, data collected after the test day were not included in the training set for that fold). The code for feature extraction and analysis is available online [37].

## Results

Models using LightGBM performed best for the population model. We used 0 as the random seed, 200 as the number of boosted trees, and 128 as the maximum tree leaves. The learning rate was chosen from {0.008, 0.01, 0.012} and the subsample ratio of columns when constructing each tree was chosen from {0.68, 0.7, 0.72}. Using this approach, models using smartphone and wearable feature data were able to correctly predict whether the next day would be a high symptom day with 73.5% accuracy (0.611 recall for the high symptom class and 0.772 AUC). This model surpassed the accuracy and performance of all 3 baseline models (Table 1).

**Table 1:** Performance of population models classifying next-day symptom class.^a^

Method	Accuracy (%)	Precision0 (%)	Recall0 (%)	F10 (%)	Precision1 (%)	Recall1 (%)	F11 (%)	Macro F1^b^ (%)	AUC (%)
Baseline1: majority class	64.5	64.5	100.0	78.4	0.0	0.0	0.0	39.2	50.0
Baseline2: random weighted classifier	54.1	64.4	64.4	64.4	35.5	35.5	35.5	50.0	50.0
Baseline3: decision tree with nonsensor features	67.5	75.5	73.3	74.4	54.0	57.0	55.5	64.9	65.1
LightGBM	73.5	78.9	80.4	79.7	63.2	61.1	62.2	70.9	77.2

^a^0=average or lower than average symptom burden; 1=higher than average symptom burden.

^b^Macro F1 score refers to the average of the 2 F1 scores.

**Figure 1:**
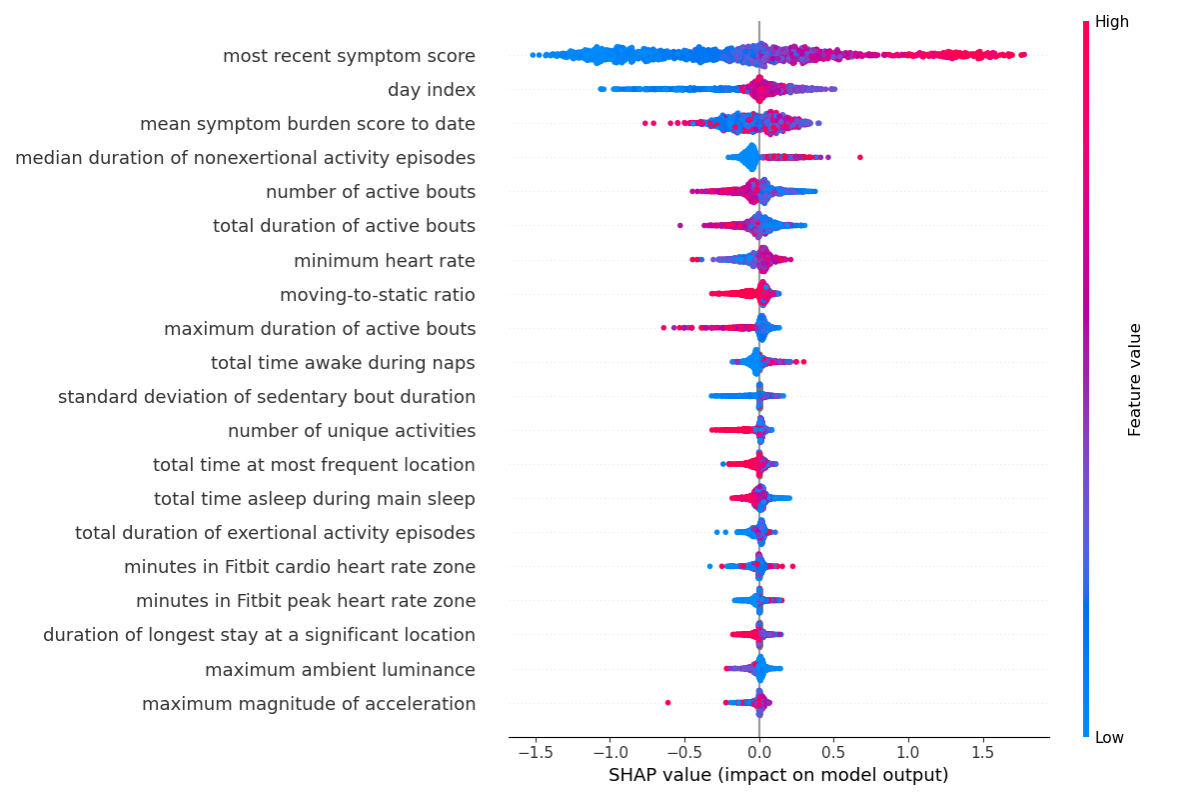
Density scatter plot showing SHapley Additive exPlanation (SHAP) values for each feature, reflecting how much impact each feature has on model output. Features with many instances in red with SHAP values greater than 0 are positively associated with symptom burden, while those with many blue instances are inversely associated with symptom burden.

The most important features included the most recent symptom burden score, days since surgery, average symptom burden score, duration of active and exertional activity bouts, minimum heart rate, number of unique activities, time spent at the most frequent location, maximum ambient lux, total duration of time awake and asleep, and total duration of the heart rate in cardio zone (70%-84% of the participant’s maximum heart rate) and peak zone (85%-100% of the participant’s maximum heart rate; Figure 1). In this plot, features with many instances in red with SHAP (SHapley Additive exPlanations) [38] value greater than 0 had a positive relationship with symptom burden (eg, longer median duration of nonexertional episodes related to high symptom burden), whereas those in blue had an inverse association (eg, shorter total duration of active bouts related to high symptom burden).

We also generated population models for diarrhea, fatigue, and pain, respectively. All steps are the same as above except for the target values. Instead of calculating the labels based on the summation of all 10 symptom ratings, diarrhea score or fatigue score or pain score is applied directly.

Like the overall symptom burden results, LightGBM models outperformed all 3 baseline models and predicted next-day diarrhea with 79.0% accuracy (AUC 83.41%), next-day fatigue with 75.8% accuracy (AUC 80.29%), and next-day pain with 79.6% accuracy (AUC 83.48%; Table 2). Location features are very important for diarrhea prediction, while step features and sleep features are very important for fatigue prediction and pain prediction, respectively. The most recent symptom burden score, days since surgery, and average symptom burden score are the most important features for all symptoms.

**Table 2:** Performance of population models classifying next-day diarrhea or fatigue or pain symptom class (1=higher than average) from wearable and smartphone sensors.

Target (symptom) and method		Accuracy (%)	Precision0 (%)	Recall0 (%)	F10 (%)	Precision1 (%)	Recall1 (%)	F11 (%)	Macro F1 (%)	AUC (%)
**Diarrhea**										
	Baseline1: majority class	67.4	67.4	100.0	80.5	0.0	0.0	0.0	40.3	50.0
	Baseline2: random weighted classifier	56.0	67.4	67.4	67.4	32.5	32.5	32.5	49.9	49.9
	Baseline3: decision tree with nonsensor features	73.2	82.0	77.2	79.5	57.9	64.9	61.2	70.3	71.0
	LightGBM	79.0	85.0	83.7	84.3	67.3	69.4	68.3	76.3	83.4
**Fatigue**										
	Baseline1: majority class	64.7	64.7	100.0	78.6	0.0	0.0	0.0	39.3	50.0
	Baseline2: random weighted classifier	54.3	64.7	64.7	64.7	35.3	35.3	35.3	50.0	50.0
	Baseline3: decision tree with nonsensor features	67.0	75.9	71.8	73.8	53.0	58.2	55.4	64.6	65.0
	LightGBM	75.8	81.2	81.5	81.4	65.9	65.5	65.7	73.5	80.3
**Pain**										
	Baseline1: majority class	70.4	70.4	100.0	82.7	0.0	0.0	0.0	41.3	50.0
	Baseline2: random weighted classifier	58.4	70.5	70.4	70.4	29.6	29.6	29.6	50.0	50.0
	Baseline3: decision tree with nonsensor features	74.4	82.4	81.0	81.7	56.5	58.8	57.6	69.7	69.9
	LightGBM	79.6	85.7	85.3	85.5	65.4	66.0	65.7	75.6	83.5

## Discussion

The purpose of this prospective longitudinal study was to evaluate passive smartphone and wearable sensor features as predictors of symptom burden in perioperative patients undergoing pancreatic surgery. Results suggest that machine learning models developed using mobile sensor data were more accurate than non–sensor-based baseline models in predicting whether the next-day patient-reported overall symptom burden would be higher than average for that patient. The most important features for symptom prediction included features related to physical activity, heart rate, and location. Models also accurately predicted next-day diarrhea, fatigue, and pain, although the most important features in each model differed across specific symptoms.

This work contributes to a small but growing literature investigating associations between consumer mobile sensors and clinical outcomes in oncology [27]. Similar to studies of patients undergoing chemotherapy [39] and hematopoietic cell transplant [40], features related to physical activity were most strongly related to fluctuations in physical symptom severity. Feature importance revealed that these were not simple features such as daily step counts but rather features reflecting patterns of activity and included measurements from both wearable Fitbit devices (eg, number, total duration, and maximum duration of active bouts) and smartphones (eg, duration of nonexertional episodes from phone accelerometer, number of unique activities recognized). Heart rate and sleep features were also important, suggesting that future work in this area should consider using wearable devices that enable collection of 24-hour behavioral and physiological data and examination of circadian rest-activity rhythms previously linked to outcomes in patients with cancer [41].

Because wearable and smartphone sensor data can be collected continuously as patients go about their daily lives, requiring minimal effort or attention from patients or their caregivers, mobile sensing offers an opportunity for long-term remote patient monitoring over months or years of cancer treatment and survivorship. This study supports the feasibility of collecting mobile sensor data, even from patients who are seriously ill during times of acute sickness and recovery. Despite undergoing invasive surgery and (for most patients) grappling with one of the deadliest cancer diagnoses, over 80% of participants had sufficient sensor data for analyses. This is also noteworthy given that the average age of patients was over 65 and that, as these data were collected in 2017, participants varied considerably in their comfort and familiarity with mobile technology.

Although models trained on past mobile sensor data outperformed baseline models, model performance still may not be adequate for clinical implementation. For example, recall of the high overall symptom burden class (when timely clinical action would be needed) was only 61%, meaning nearly 40% of high symptom days would be missed by our model. This may be due in part to the relatively small sample and data set, the use of study-provided (rather than personal) smartphones, or the powerful effect of major abdominal surgery and prolonged hospitalization on patient symptom profiles as well as behavior. Future studies with larger samples that collect data using their own personal devices over a period with less dramatic shifts in symptoms and behavior may yield better model performance. In future studies with larger data sets more robust to class imbalance, setting a higher threshold for severe symptoms requiring care provider attention or intervention may also result in more clinically useful models. Regardless, mobile sensor data may be a useful complement to patient-reported symptom data, allowing for a more personalized and adaptive delivery of symptom ratings when behavioral fluctuations are detected, reducing patient burden and improving early capture of worsening side effects and symptoms. Predictive models based on sensor and patient-reported data could also be used to deliver symptom self-management instructions to patients, an approach demonstrated to benefit patients undergoing pancreatic cancer surgery [42].

Given the small data set, we focused on building population models that used data from all other participants, which also may have constrained model performance. Because each participant had on average only 30 rows of data, individual models were unstable, but with more training data could be useful in learning patterns based on each participant’s behavior and its relationship to symptoms and developing more accurate predictions. Developing models based on similar subgroups of participants (based on demographic, clinical, or behavioral factors) could be a useful approach for future work and could yield superior results to a single population model.

Strengths of the study include longitudinal sensor data collection over a wide perioperative window, from presurgery to 60 days after discharge following pancreatic surgery. We considered a wide range of features from both wearable and smartphone sensors and examined prediction of next-day overall symptom burden as well as next-day pain, fatigue, and diarrhea specifically. Our models were also trained on past data only so that we could evaluate how well models could perform if implemented in real-world clinical settings.

This study suggests that digital biomarkers may be useful in predicting patient-reported symptom burden during cancer treatment. In an ongoing study, we are following up on this work by collecting 3 months of smartphone and wearable sensor data as well as daily symptom reports from a large sample of patients undergoing outpatient chemotherapy. With a larger outpatient sample using their own smartphones, we hope to improve upon the models developed here and to use real-time next-day symptom predictions to deliver more timely and personalized symptom management support.

## Abbreviations

AUC: area under the ROC curve
LightGBM: light gradient boosting machine
PRO: patient-reported outcome
RAPIDS: Reproducible Analysis Pipeline for Data Streams
ROC: receiver operating characteristic
SHAP: SHapley Additive exPlanations
SVM SMOTE: support vector machine synthetic minority over-sampling technique

## References

[1] Henry DH, Viswanathan HN, Elkin EP, Traina S, Wade S, Cella D (2008). Symptoms and treatment burden associated with cancer treatment: results from a cross-sectional national survey in the U.S. Support Care Cancer.

[2] Reilly CM, Bruner DW, Mitchell SA, Minasian LM, Basch E, Dueck AC, Cella D, Reeve BB (2013). A literature synthesis of symptom prevalence and severity in persons receiving active cancer treatment. Support Care Cancer.

[3] Cleeland CS, Zhao F, Chang VT, Sloan JA, O'Mara AM, Gilman PB, Weiss M, Mendoza TR, Lee J, Fisch MJ (2013). The symptom burden of cancer: Evidence for a core set of cancer-related and treatment-related symptoms from the Eastern Cooperative Oncology Group Symptom Outcomes and Practice Patterns study. Cancer.

[4] Hensing T, Cella D, Yount S (2005). The impact of ECOG performance status on quality of life symptoms in patients with advanced lung cancer. JCO.

[5] West HJ, Jin JO (2015). JAMA Oncology Patient Page. Performance Status in Patients With Cancer. JAMA Oncol.

[6] Atkinson TM, Ryan SJ, Bennett AV, Stover AM, Saracino RM, Rogak LJ, Jewell ST, Matsoukas K, Li Y, Basch E (2016). The association between clinician-based common terminology criteria for adverse events (CTCAE) and patient-reported outcomes (PRO): a systematic review. Support Care Cancer.

[7] Fromme EK, Eilers KM, Mori M, Hsieh Y, Beer TM (2004). How accurate is clinician reporting of chemotherapy adverse effects? A comparison with patient-reported symptoms from the Quality-of-Life Questionnaire C30. J Clin Oncol.

[8] Schneider S, Stone AA (2015). Ambulatory and diary methods can facilitate the measurement of patient-reported outcomes. Qual Life Res.

[9] Anderson M (2019). Mobile Technology and Home Broadband 2019.

[10] Falchook AD, Tracton G, Stravers L, Fleming ME, Snavely AC, Noe JF, Hayes DN, Grilley-Olson JE, Weiss JM, Reeve BB, Basch EM, Chera BS (2016). Use of mobile device technology to continuously collect patient-reported symptoms during radiation therapy for head and neck cancer: A prospective feasibility study. Adv Radiat Oncol.

[11] Weaver A, Young AM, Rowntree J, Townsend N, Pearson S, Smith J, Gibson O, Cobern W, Larsen M, Tarassenko L (2007). Application of mobile phone technology for managing chemotherapy-associated side-effects. Ann Oncol.

[12] Kearney N, McCann L, Norrie J, Taylor L, Gray P, McGee-Lennon M, Sage M, Miller M, Maguire R (2009). Evaluation of a mobile phone-based, advanced symptom management system (ASyMS) in the management of chemotherapy-related toxicity. Support Care Cancer.

[13] Judson TJ, Bennett AV, Rogak LJ, Sit L, Barz A, Kris MG, Hudis CA, Scher HI, Sabattini P, Schrag D, Basch E (2013). Feasibility of long-term patient self-reporting of toxicities from home via the Internet during routine chemotherapy. J Clin Oncol.

[14] Min YH, Lee JW, Shin Y, Jo M, Sohn G, Lee J, Lee G, Jung KH, Sung J, Ko BS, Yu J, Kim HJ, Son BH, Ahn SH (2014). Daily collection of self-reporting sleep disturbance data via a smartphone app in breast cancer patients receiving chemotherapy: a feasibility study. J Med Internet Res.

[15] Harari GM, Lane ND, Wang R, Crosier BS, Campbell AT, Gosling SD (2016). Using Smartphones to Collect Behavioral Data in Psychological Science: Opportunities, Practical Considerations, and Challenges. Perspect Psychol Sci.

[16] Andrews S, Ellis DA, Shaw H, Piwek L (2015). Beyond Self-Report: Tools to Compare Estimated and Real-World Smartphone Use. PLoS One.

[17] Mohr DC, Zhang M, Schueller SM (2017). Personal Sensing: Understanding Mental Health Using Ubiquitous Sensors and Machine Learning. Annu Rev Clin Psychol.

[18] Friedenreich CM, Neilson HK, Farris MS, Courneya KS (2016). Physical Activity and Cancer Outcomes: A Precision Medicine Approach. Clin Cancer Res.

[19] Kloter E, Barrueto K, Klein SD, Scholkmann F, Wolf U (2018). Heart Rate Variability as a Prognostic Factor for Cancer Survival - A Systematic Review. Front Physiol.

[20] Li Y, Cai S, Ling Y, Mi S, Fan C, Zhong Y, Shen Q (2019). Association between total sleep time and all cancer mortality: non-linear dose-response meta-analysis of cohort studies. Sleep Med.

[21] Saeb S, Zhang M, Karr CJ, Schueller SM, Corden ME, Kording KP, Mohr DC (2015). Mobile Phone Sensor Correlates of Depressive Symptom Severity in Daily-Life Behavior: An Exploratory Study. J Med Internet Res.

[22] Doryab A, Frost M, Faurholt-Jepsen M, Kessing L, Bardram J (2014). Impact factor analysis: combining prediction with parameter ranking to reveal the impact of behavior on health outcome. Pers Ubiquit Comput.

[23] Frost M, Doryab A, Faurholt-Jepsen M, Kessing L, Bardram J (2013). Supporting disease insight through data analysis: refinements of the monarca self-assessment system. UbiComp '13: Proceedings of the 2013 ACM international joint conference on Pervasive and ubiquitous computing.

[24] Grünerbl A, Muaremi A, Osmani V, Bahle G, Ohler S, Tröster G, Mayora O, Haring C, Lukowicz P (2015). Smartphone-based recognition of states and state changes in bipolar disorder patients. IEEE J Biomed Health Inform.

[25] Wang R, Aung M, Abdullah S (2016). CrossCheck: toward passive sensing and detection of mental health changes in people with schizophrenia.

[26] Bae S, Ferreira D, Suffoletto B, Puyana JC, Kurtz R, Chung T, Dey AK (2017). Detecting Drinking Episodes in Young Adults Using Smartphone-based Sensors. Proc. ACM Interact. Mob. Wearable Ubiquitous Technol.

[27] Low CA (2020). Harnessing consumer smartphone and wearable sensors for clinical cancer research. NPJ Digit Med.

[28] Panda N, Solsky I, Huang EJ, Lipsitz S, Pradarelli JC, Delisle M, Cusack JC, Gadd MA, Lubitz CC, Mullen JT, Qadan M, Smith BL, Specht M, Stephen AE, Tanabe KK, Gawande AA, Onnela J, Haynes AB (2020). Using Smartphones to Capture Novel Recovery Metrics After Cancer Surgery. JAMA Surg.

[29] Low CA, Bovbjerg DH, Ahrendt S, Choudry MH, Holtzman M, Jones HL, Pingpank JF, Ramalingam L, Zeh HJ, Zureikat AH, Bartlett DL (2018). Fitbit step counts during inpatient recovery from cancer surgery as a predictor of readmission. Ann Behav Med.

[30] Strobel O, Neoptolemos J, Jäger D, Büchler MW (2019). Optimizing the outcomes of pancreatic cancer surgery. Nat Rev Clin Oncol.

[31] Ferreira D, Kostakos V, Dey A (2015). AWARE: mobile context instrumentation framework. Frontiers in ICT.

[32] Reeve BB, Mitchell SA, Dueck AC, Basch E, Cella D, Reilly CM, Minasian LM, Denicoff AM, O'Mara AM, Fisch MJ, Chauhan C, Aaronson NK, Coens C, Bruner DW (2014). Recommended patient-reported core set of symptoms to measure in adult cancer treatment trials. J Natl Cancer Inst.

[33] Cleeland CS, Mendoza TR, Wang XS, Chou C, Harle MT, Morrissey M, Engstrom MC (2000). Assessing symptom distress in cancer patients: the M.D. Anderson Symptom Inventory. Cancer.

[34] Tedesco S, Sica M, Ancillao A, Timmons S, Barton J, O'Flynn B (2019). Validity Evaluation of the Fitbit Charge2 and the Garmin vivosmart HR+ in Free-Living Environments in an Older Adult Cohort. JMIR Mhealth Uhealth.

[35] Vega J, Li M, Aguillera K, Goel N, Joshi E, Durica KC, Kunta AR, Low CA RAPIDS: Reproducible Analysis Pipeline for Data Streams Collected with Mobile Devices. J Med Internet Res Preprints.

[36] RAPIDS.

[37] Low C, Li M, Vega J, Durica K, Ferreira D, Tam V, Hogg M, Zeh H, Doryab A, Dey AK (2021). carissalow/rhythms-population: v1.0.1.

[38] Lundberg SM, Erion G, Chen H, DeGrave A, Prutkin JM, Nair B, Katz R, Himmelfarb J, Bansal N, Lee S (2020). From Local Explanations to Global Understanding with Explainable AI for Trees. Nat Mach Intell.

[39] Low CA, Dey AK, Ferreira D, Kamarck T, Sun W, Bae S, Doryab A (2017). Estimation of Symptom Severity During Chemotherapy From Passively Sensed Data: Exploratory Study. J Med Internet Res.

[40] Bennett AV, Reeve BB, Basch EM, Mitchell SA, Meeneghan M, Battaglini CL, Smith-Ryan AE, Phillips B, Shea TC, Wood WA (2016). Evaluation of pedometry as a patient-centered outcome in patients undergoing hematopoietic cell transplant (HCT): a comparison of pedometry and patient reports of symptoms, health, and quality of life. Qual Life Res.

[41] Innominato PF, Komarzynski S, Palesh OG, Dallmann R, Bjarnason GA, Giacchetti S, Ulusakarya A, Bouchahda M, Haydar M, Ballesta A, Karaboué A, Wreglesworth NI, Spiegel D, Lévi FA (2018). Circadian rest-activity rhythm as an objective biomarker of patient-reported outcomes in patients with advanced cancer. Cancer Med.

[42] Gustavell T, Sundberg K, Langius-Eklöf A (2020). Using an Interactive App for Symptom Reporting and Management Following Pancreatic Cancer Surgery to Facilitate Person-Centered Care: Descriptive Study. JMIR Mhealth Uhealth.

